# Bilateral Obstructive Uropathy Secondary to Giant Periureteral Diverticulum

**DOI:** 10.1155/2013/747412

**Published:** 2013-10-22

**Authors:** Alberto Hernando Arteche, Luis Alberto San Jose Manso, Carlos Olivier Gomez, Angel Nellyt Silmi Moyano

**Affiliations:** ^1^Department of Urology, Hospital Central de la Defensa “Gómez Ulla”, Madrid, Spain; ^2^Department of Urology, Hospital Infanta Sofia, Madrid, Spain; ^3^Department of Urology, Hospital de La Princesa, Madrid, Spain; ^4^Department of Urology, Hospital Clínico San Carlos, Madrid, Spain

## Abstract

Bladder diverticula are herniations of the mucosa through the fibers of the bladder muscle connected by necks of variable amplitude. They are often asymptomatic, although they may lead to complications that require a surgical therapeutic approach. We report the case of a patient with bilateral obstructive uropathy secondary to a giant periureteral diverticulum that was treated by diverticulectomy and reimplantation of the left ureter in the bladder.

## 1. Introduction

A bladder diverticulum is a herniation of the bladder or urothelial mucosa through the detrusor muscle fibers, resulting in a hernia sac connected to the bladder more or less extensively by the diverticular neck. Diverticula are usually asymptomatic and are sometimes diagnosed by ultrasound. However, further endoscopic and imaging examinations (excretory urography (IVU) that combines retrograde cystography, computed urotomography (uro-CT), or magnetic resonance imaging (MRI)) are required for the proper characterisation of the diverticulum before considering surgical treatment. In rare cases, diverticula present difficulties in differential diagnosis and can be confused with pseudodiverticula, urachal diverticulum, or seminal vesicle cysts, among other conditions. The most frequent complications are recurrent infections, calcifications, malignant disease, and the compression of adjacent organs; surgical treatment may be indicated in any of these cases.

## 2. Clinical Case

**Figure 1:**
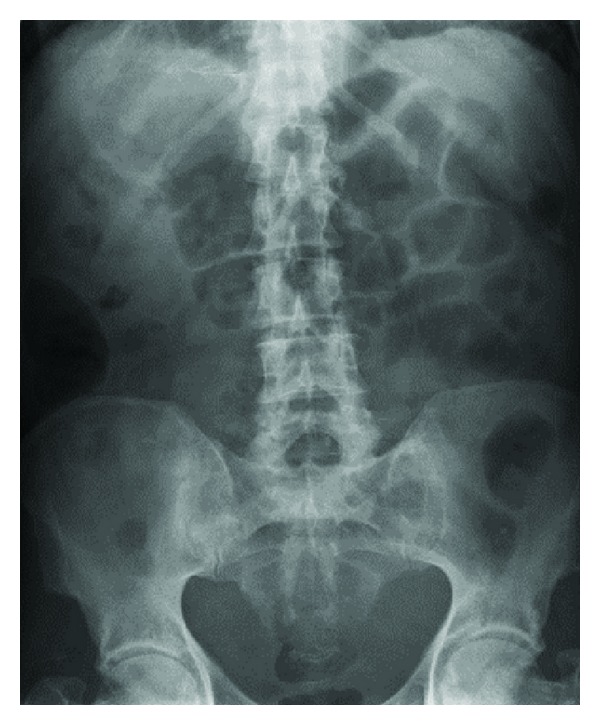
Plain abdominal radiography.

**Figure 2:**
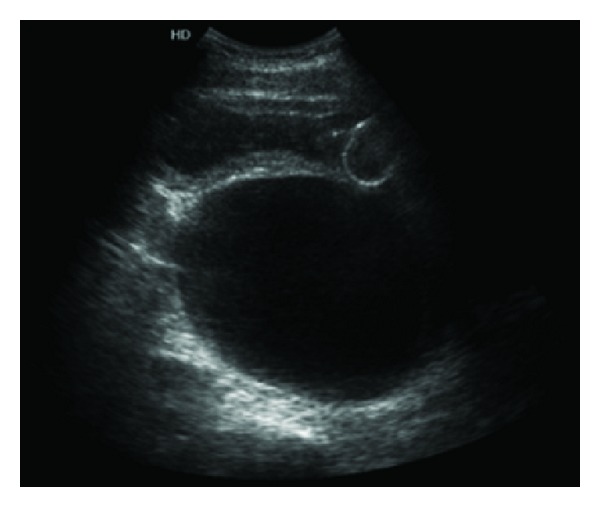
Abdominal ultrasound: large cystic image posterior to bladder with an approximate volume of 780 cc.

A 70-year-old man with a history of hypertension treated with enalapril and atrial fibrillation treated with digoxin, antiplatelet therapy, and aspirin came to the emergency department with significant dysuria; increased urinary frequency; decreased urinary force and global diuresis; and lumbar, hypogastric, and penile pain lasting for 2 days. A physical examination revealed diffuse abdominal pain and tenderness deep in the hypogastrium with positive bowel sounds and a DRE prostate volume I/IV classified as adenomatous and not suspicious for malignancy. Blood testing showed 3.1 mg/dL creatinine (Cr), 56 mg/dL urea, 139 mmol/L Na^+^, 3.7 mmol/L K^+^, and a white blood cells (WBC) count of 8750 (71% neutrophils). Plain abdominal radiography (Figure 1) revealed no relevant changes in the region of interest, and an ultrasound (Figure 2) showed bilateral obstructive uropathy, a bladder with signs of struggle, and a retroperitoneal cyst with a volume of 780 cc.

A diagnosis was made of chronic urine retention, and a bladder catheter was placed, obtaining 150 cc of urine. Within hours, the patient presented with oliguria and renal function further deteriorated to 5.5 mg/dL Cr.

**Figure 3:**
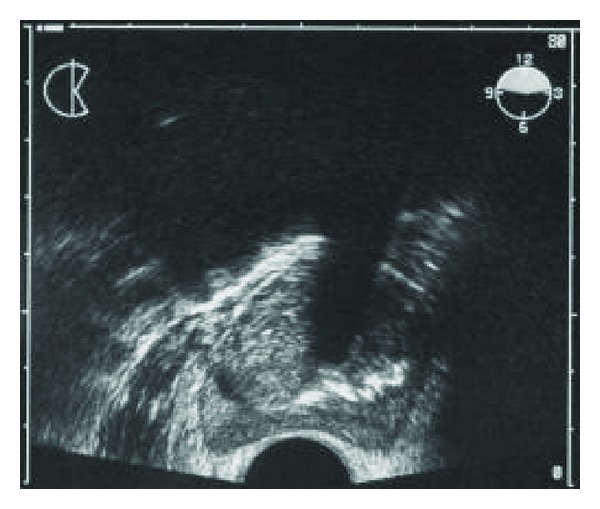
Transrectal ultrasound: hypoechoic lesion posterior and cranial to the prostate.

Eco-TR (Figure 3) showed a large anechoic lesion in the theoretical position of the left seminal vesicle displacing and compressing the bladder, and cystoscopy showed an elevation of trigone secondary to extrinsic compression that prevented the identification of the ureteral meatus and the prostate Shivers II.

**Figure 4:**
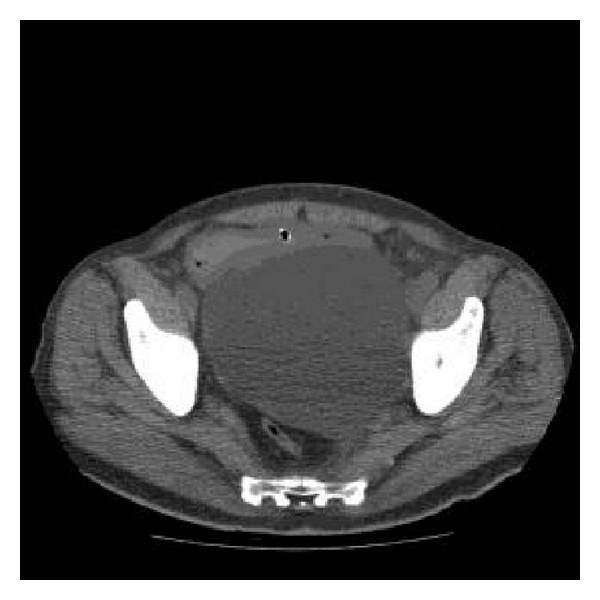
Abdominal CT scan: retrovesical 14 × 14 × 12 cm cystic mass and bilateral obstructive uropathy.

**Figure 5:**
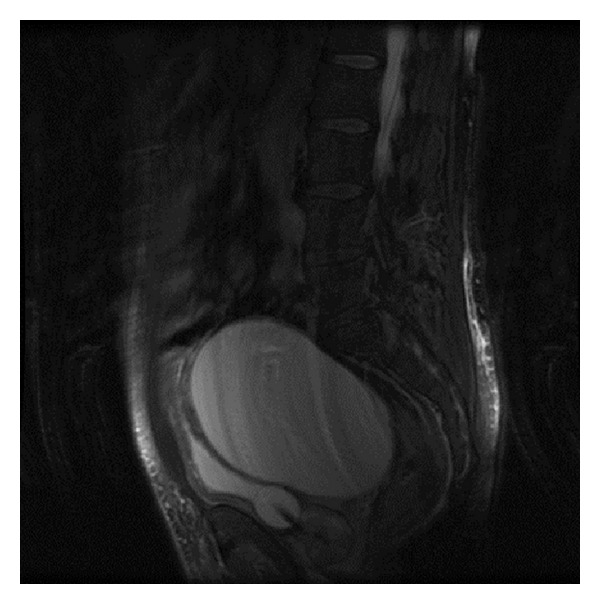
Uro-NMR: retrovesical 12 × 14 cm mass cranial to the prostate and seminal vesicles and without communication with the bladder.

The persistent worsening of renal function (7.6 mg/dL Cr) led to urinary diversion by percutaneous nephrostomy placement and performance of TAC (Figure 4) to visualise the retrovesical cystic mass, which indicated bilateral obstructive uropathy. Subsequently a uro-NMR study (Figure 5) showed a retrovesical 12 × 14 cm cystic mass above the bladder with no communication with the bladder or dependence on other nearby structures. Given these findings, surgical exploration was chosen, during which a large retrovesical cyst was released to the retrotrigone, where both ureters were identified and located in the left wall. The cyst was opened and found to be consistent with a left periureteral diverticulum, with the mouth of the diverticulum identified near the left ureteral orifice. The diverticulum was resected, and left extravesical ureteral reimplantation was performed with a double-J catheter. The patient had a good outcome and tolerated the removal of the nephrostomy and catheter. Renal function recovered, with a high Cr reading of only 1.3 mg/dL.

## 3. Discussion 

Bladder diverticulum is a herniation of the bladder mucosa through the muscular fibers, resulting in a cavity connected to the bladder through a narrow neck. The diverticulum wall is characterised by the absence of a muscle layer and thus contractile function, with the result that emptying of the diverticulum will occur in a passive manner dependent on the neck of the diverticulum [1]. This condition can be congenital or acquired. The diagnosis is made early in childhood, and diverticula are usually solitary and occur more frequently in males [2]. Diverticula are usually caused by a weakness of the ureterovesical junction secondary to a developmental abnormality, although in exceptional cases they may be part of a connective tissue disorder such as Ehlers-Danlos syndrome [3], Williams syndrome, and Menkes syndrome or Tricholipodystrophy [4]. Acquired or secondary diverticula occur more frequently in patients with an intravesical urinary obstruction (e.g., benign prostatic hypertrophy, prostate cancer, urethral strictures, and posterior urethral valves) or vesico-sphincter dyssynergia [5]. The first consequence of a diverticulum is a compensatory hypertrophy of the detrusor muscle, leading to the typical trabecula, or columns, leaving the cyst, as well as small areas of weakness, or cells that give rise to the diverticulum. These lesions are most often formed at the weakest points of the bladder wall such as the insertion of the urachus or posterolateral position to the ureteral orifices [5], with location in the trigone being an exception. The vast majority of diverticula are asymptomatic, and a high percentage of cases are discovered incidentally when performing an imaging test or endoscopy. Characteristic diverticulum symptoms include mictionation in two phases (for delayed emptying of the urine retained in the diverticulum) and the sensation of a weight or lump in the lower abdomen that worsens with bladder filling [6]. Among the most frequently reported complications are recurrent urinary tract infections (up to 70%) [7], malignant intradiverticular tumors (0.8 to 13.5%) [2, 6], vesicoureteral reflux [4], ureteral obstruction (5–15%) [6, 7], and spontaneous rupture [8, 9]. Other rare complications reported in the literature for patients with giant diverticula include presentation with an inferior vena cava syndrome [7], subacute intestinal obstruction [4, 10], recurrent acute urinary retention [11], or exceptionally bilateral hydronephrosis [5], as in our case. New imaging techniques have largely replaced the classical retrograde urography and cystography as tools for completely characterising a diverticulum and planning appropriate surgical treatment. Uro-CT is currently the preferred technique for the study of bladder diverticula due to its effectiveness, accessibility, and ability to perform three-dimensional reconstructions with the multislice CT. MRI can also identify the diverticular neck but adds nothing new to the information provided by the uro-TC [6]. Treatment of a bladder diverticulum is indicated in cases that are symptomatic or that produce any of the complications listed above. Commonly used techniques include establishing a diverticular neck opening endoscopically or extravesical diverticulectomu or intravesical diverticulectomy. Cases of malignant neoplasm with involvement of perivesicular fat may require radical cystectomy, and in some cases a ureteral reimplantation is required if the ureter is affected. Obstructive disease must be treated based on the acquired diverticula, and treatment normally consists of transurethral resection of the prostate or open adenomectomy [2, 6].
